# A Review of Particle Size Analysis with X-ray CT

**DOI:** 10.3390/ma16031259

**Published:** 2023-02-01

**Authors:** Julia G. Behnsen, Kate Black, James E. Houghton, Richard H. Worden

**Affiliations:** 1 School of Engineering, University of Liverpool, Liverpool L69 3GH, UK; 2 Department of Earth, Ocean and Ecological Science, University of Liverpool, Liverpool L69 3GH, UK

**Keywords:** X-ray computed tomography, particle size distribution, particle shape measurements, powder sample preparation

## Abstract

Particle size and morphology analysis is a problem common to a wide range of applications, including additive manufacturing, geological and agricultural materials’ characterisation, food manufacturing and pharmaceuticals. Here, we review the use of microfocus X-ray computed tomography (X-ray CT) for particle analysis. We give an overview of different sample preparation methods, image processing protocols, the morphology parameters that can be determined, and types of materials that are suitable for analysis of particle sizes using X-ray CT. The main conclusion is that size and shape parameters can be determined for particles larger than approximately 2 to 3 μm, given adequate resolution of the X-ray CT setup. Particles composed of high atomic number materials (Z > 40) require careful sample preparation to ensure X-ray transmission. Problems occur when particles with a broad range of sizes are closely packed together, or when particles are fused (sintered or cemented). The use of X-ray CT for particle size analysis promises to become increasingly widespread, offering measurements of size, shape, and porosity of large numbers of particles within one X-ray CT scan.

## 1. Introduction

**Figure 1:**
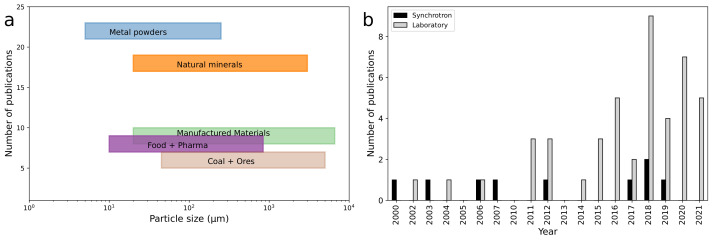
Diagrams summarising the history of use of X-ray CT for particle size characterisation. (**a**) The number of publications for different material categories. The category “natural minerals” contains studies that used naturally occurring minerals, such as naturally occurring sand [6,11,14,27,49,56,59] or crushed granite [42], while the category “manufactured materials” contains artificially-created particles, such as beads made from glass [1], acrylic [5], gypsum [17], or ceramic [39]. The particle size ranges measured within each category are shown on the x-axis. (**b**) The number of publications utilising particle size characterisation with X-ray CT since the year 2000, split into use of synchrotron vs. laboratory X-ray sources.

**Figure 2:**
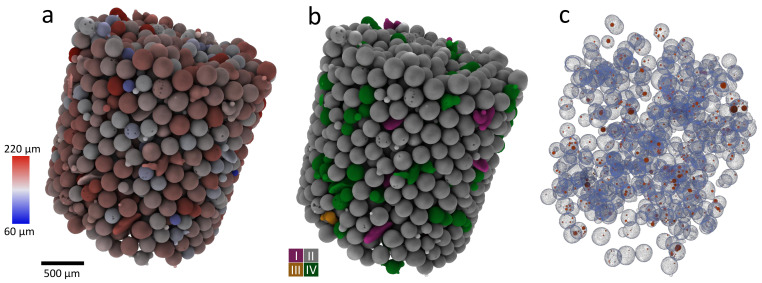
Micrometre-size glass bead quantification with X-ray CT. (**a**) Glass beads coloured according to equivalent diameter from blue (60 μm) to red (220 μm). (**b**) The same glass beads coloured according to Zingg’s [66] shape classification (discs (I): purple, spheres (II) grey, blades (III): orange, rods (IV): green). (**c**) Only beads with internal pores shown (beads in blue, pores in red).

Over the last 20 years (Figure 1a), more than 60 publications [1,2,3,4,5,6,7,8,9,10,11,12,13,14,15,16,17,18,19,20,21,22,23,24,25,26,27,28,29,30,31,32,33,34,35,36,37,38,39,40,41,42,43,44,45,46,47,48,49,50,51,52,53,54,55,56,57,58,59,60,61] have utilised microfocus X-ray-computed tomography (also known as micro-CT, μX-ray CT, and XCT) for the analysis of particles in the range of micro- to millimetres. The applications derive from such diverse fields as additive manufacturing [8,19,21,23,28,31,32,33,34,35,37,40,43,45,46,52,58,61], granular packing studies [1,5,11,17,49,59,62], food processing [12,20,24], and pharmaceutical applications [10,51,55,57], because they all involve finely divided materials and benefit from particle size characterisation. The ease of sample preparation (Section 2.1) and the amount of information available for each single particle (Section 2.5) are other reasons for the breadth of use of X-ray CT. The 3D size, morphology, internal porosity, and the position of a given particle in the granular assembly are types of information that are available from a single scan (Figure 2). Due to the non-destructive nature of X-ray CT imaging, repeated scans of the same sample after an intervention such as loading [11,16,49,56] or heating [3] are possible and allow for time-lapse (4D) studies of changes in the assembly [53]. The digital data, collected with each scan, can feed directly into computed models about the particles and their behaviour in the granular assembly [27,47,48,54]. After early use of synchrotron beam lines [1,3,6,7,14,28,33,34,46], the emergence of laboratory X-ray CT instruments [2,4,8,9,10,11,12,13,18,20,21,22,23,24,25,26,27,30,31,32,36,37,38,39,40,41,42,43,44,45,47,48,49,50,51,52,54,55,57,58,59,60] has made the technique more widely accessible (Figure 1b).

This review summarises studies that have utilised X-ray CT for particle size (and morphology) quantification. In this context, a particle is a small (micro- to millimetre scale) rigid body. The distribution of particle properties, such as size and shape over a large number of similar particles, rather than the properties of a specific single particle, is typically of interest to the analysis. In different research fields, individual particles might also be called grains; similarly, a collection of particles may be known as a granular assembly, a powder, or bulk material. We will refer to “loose particles” for a collection of particles that have no strong binding forces or adhesion between them and will flow freely if not constrained in a container. To be clear, terms such as grain size analysis or powder analysis are equally used for the same methodology in different scientific disciplines.

Reviews of the general use of X-ray CT for the broad field of materials research [63], and specifically additive manufacturing [64,65], have recently been published, but these did not focus on particle characterisation. A detailed description of the experimental approach to particle size analysis that was employed by a single laboratory is available [52], but it excludes methods used at other institutions. The use of X-ray CT for the time-lapse study of particulate systems has also been recently published [53], but omitted the basic characterisation of the loose particles, which is our focus here.

The aim of this review is to address particle characterisation using X-ray CT, answering the following specific questions:

- What materials and particle sizes have been analysed with this method?
- What are the options for sample preparation, and are they influenced by the particles to be measured?
- What influence does the image processing methodology have on the results?
- Where are the limits of the method in terms of material suitability and particle size range?

We intend that this review will serve as a guide for researchers and others new to particle analysis with X-ray CT, who are considering using this method for their own samples. A further objective of this review is to develop a common language related to particle size analysis that can be adopted across different disciplines.

### 1.1. History of Particle Analysis with X-ray CT

X-ray computed tomography, the method of taking a series of X-ray projection images around the object of interest, and computing a tomographic dataset (a 3D image) from the result, has become a major diagnostic tool since the first commercial medical scanner was built in 1973 [67]. Since then, the development of industrial scanners [68] and improvements to microfocus X-ray sources and detectors to enable micron and submicron-range resolution [69,70] (often termed micro-CT) opened the door for the characterisation of a wide range of materials, including particles of the μm to mm range.

**Figure 3:**
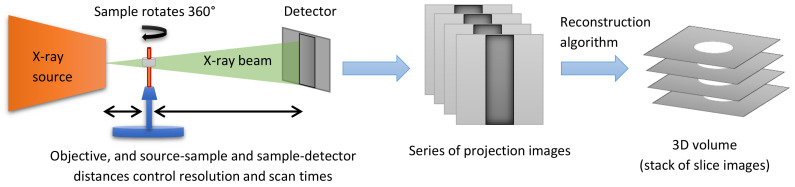
Schematic of a typical laboratory microfocus X-ray CT acquisition system, showing the source, sample with sample holder, and flat-panel detector. Source-sample and sample-detector distances affect image magnification. A series of projection images is captured while rotating the sample, commonly over 360${}^{\circ }$, which is then reconstructed into the slice images that form the 3D data volume.

A simple schematic diagram of the X-ray CT data acquisition system is presented in Figure 3. More comprehensive descriptions are available elsewhere [63,68], but a brief summary is included here. In laboratory X-ray CT instruments, typically a sample is positioned upright between the microfocus X-ray source and the detector. The X-ray source usually allows variation of the maximum energy of the X-ray beam, with common maximum energies up to 225 keV. Beam filters can be used to remove the low-energy component of the X-ray spectrum and achieve a higher relative transmission through the sample. The X-ray beam is transmitted through the sample and attenuated by the materials that compose it. The attenuation of the beam is revealed in the intensity distribution recorded by the detector for each projection image. The detector consists of a scintillator and a photon counting device. Flat panel detectors are most commonly used, but some high-resolution instruments utilise multiple objectives with different optical magnification instead (typically called X-ray microscopes). A series of projection images are captured while rotating the sample. While a rotation of 180${}^{\circ }$ is sufficient for parallel-beam geometries, such as in synchrotrons, cone-beam laboratory instruments generally require a rotation over 360${}^{\circ }$ [71]. The projection images are converted into a stack of slice images by a reconstruction algorithm, typically a variation of the FDK-algorithm for divergent (cone) beam systems [72]. The study of small particles with X-ray CT started in 2000, when the individual positions of 63 μm diameter glass beads were determined at beamline 20-ID at the Advanced Photon Source, USA, to study 3D granular packing [1]. In the first decade of the millennium, approximately half of the published studies were undertaken at synchrotron beamlines; however, the increasing availability of laboratory-based X-ray CT instruments has meant that, in the last 10 years, 90% of the published studies were carried out using laboratory systems (Figure 1b). Laboratory-based X-ray CT systems are typically easier to use and timelier to access than synchrotron beamlines, and they are also easily available for commercial companies to carry out their own testing, which is an advantage for regular quality control of, for example, manufacturing feedstocks, products or food powders.

### 1.2. Summary of Materials Examined

X-ray CT methods are suitable for a broad range of materials, and a summary of those particles characterised with this method is given in Figure 1a. The characterisation of metal powders is of great interest for powder-based additive manufacturing (AM) processes, where powder properties such as particle size distribution and particle shape affect the flow and spreadability of the powder [73]. Imperfections, such as pores inside powder particles, or contamination of the powder with particles of a different material, are also of great interest since they can affect the strength of the final build-part [74]. Metal powders analysed by X-ray CT, of interest to the AM industry, include titanium alloys [8,21,28,31,33,34,46,52,58,64], steel [40,43,46,61] and nickel-based alloy [37]. These studies aimed to evaluate the quality and suitability of a powder, for example, after several rounds of recycling, or of powder particles made by different production processes [8,34,37]. Other objectives have included understanding if the porosity inside the powder particles transfers into the build part, or how particles change during sintering [3] or compaction [16].

X-ray tomography has been used to study geological material, mainly quartz-rich sand [9,11,13,18,49,50,56], but also ores and coal [4,7,25,38,48]. A field of interest is the behaviour of sand particles during deformation [11,13,36,49,53,56]. Other studies have been concerned with the development of image processing methods to aid with the identification, description, and tracking of particles during deformation [6,44], and often use a specific reference material, such as Caicos ooids [36], ceramic proppant [39], or industrially-made zeolite particles [44]. Related is the analysis of granular assemblies to describe pore networks and grain contacts [15,17,59]. Another field of research is the development of digital models of sand grains to virtually study particle breakage or failure modes [27,47,48].

Granulated organic materials, including foods, such as milk powder [12] or maltodextrin [24], pesticide-containing dust from seeds [20], and pharmaceutical powders (lactose, [51], hexamine [57], L-glutamic acid [55], acetylsalicyl acid [10]) have also been studied using X-ray CT.

### 1.3. Particle Size Ranges

Along with different material size classes, Figure 1a also shows the particle size ranges that have been studied. The smallest particle sizes analysed involve studies of metal powder, with successful analysis of particles as small as 5 μm to 25 μm [40]. However, it was noted that shape analysis of the smallest particles was not possible with the employed X-ray CT setup, which had a voxel (a 3D pixel) size of 2.9 μm. The largest particles studied that are included in this review are in the centimetre range [25,75]. Only a few dozen large particles fit the field of view at higher resolution, but scans of small particles, such as in metal powders, typically contain tens of thousands of particles; multiple scans along a sample can detail over 100,000 particles [19]. Most studies have been of spherical or equiaxial particles (e.g., sand grains). Needle or disk-shape particles have not been extensively examined, although dust particles, with more complex shapes, have been scanned [20], and irregular agglomerates resulting from spray fluidisation have been analysed [24]. As well as characterising the particle size and morphology, the internal porosity of particles has also been examined [21,23,58] (Figure 2c).

## 2. Scanning Protocol for Particle Size Analysis

**Figure 4:**
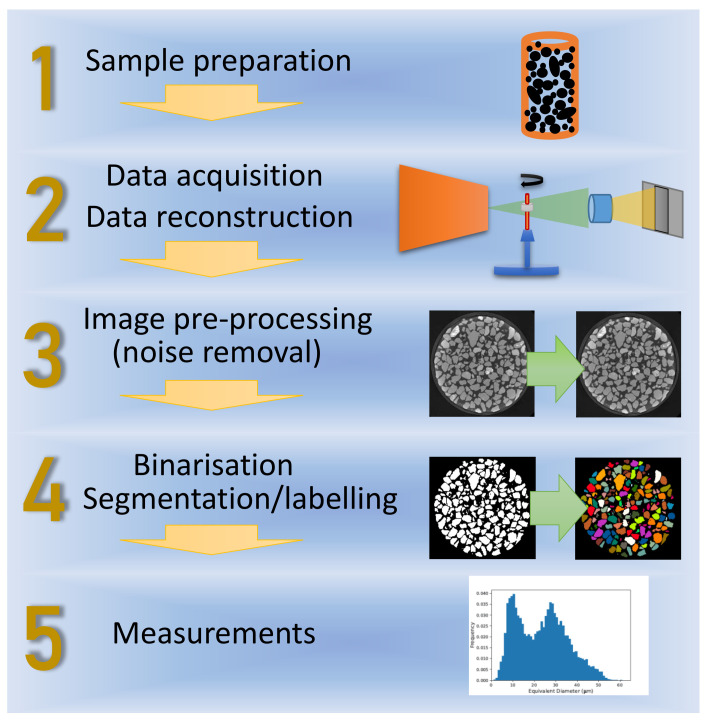
Generalised scanning and analysis workflow for particle characterisation with X-ray CT. After sample preparation (Section 2.1), the X-ray CT data are acquired and reconstructed. The resulting greyscale data are usually pre-processed, for example, for noise smoothing, before further processing. To enable quantification, each particle must be segmented (or labelled) individually (Section 2.4), which typically follows a general binarisation step that separates all particles from the surrounding medium. Once segmented, individual particle size and shape parameters can be determined (Section 2.5).

Figure 4 illustrates the steps taken to analyse particles with X-ray CT. Several variations of the individual steps have been employed; they will be compared and discussed in this section.

### 2.1. Step 1: Sample Preparation

**Figure 5:**
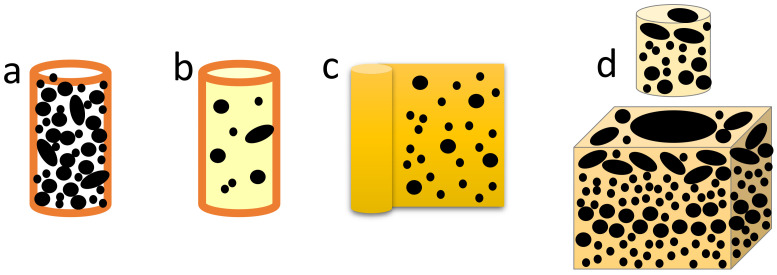
Sample preparation methods for loose particles. (**a**) Particles can be poured into a capillary. (**b**) Particles can be mixed with epoxy and cured inside a capillary. (**c**) Particles can be sprayed onto adhesive material such as wax or adhesive tape, and rolled into a cylinder. (**d**) Larger assemblies of particles can be infused with resin and then cored to obtain a smaller cylindrical sample.

In a typical laboratory X-ray CT scanner, the ideal sample is cylindrical and positioned upright with the base securely fixed (Figure 3). To enable scanning of powders, the loose particles must be held in a form that is compatible with this geometry. Four common sample preparation methods, discussed below, are illustrated in Figure 5.

The simplest preparation method is to pour loose particles into a capillary, or similar cylindrical container (Figure 5a) [8,20,21,35,51,55]. The diameter of the capillary is adjusted to the particle size to ensure enough particles are in the field of view. Both full field of view (full diameter of the capillary) and scans of an internal region of the capillary are possible to further adjust the resolution. This method is suitable for a large range of materials; additional steps at the image processing stage typically are later needed to separate the touching particles into individual ones (see Section 2.4).

To avoid the need to separate the particles with image processing steps, particles have also been dispersed in a containing medium at the sample preparation stage. One method is to mix the powder with a viscous epoxy and let this cure either inside a capillary (Figure 5b) [7,19,31,32,33,43,52,58,61] or as a block, which can then be cored (Figure 5d) [18,23]. The downside of this method is that it takes longer to prepare the sample than pouring loose material into a capillary and it is not suitable for all materials (e.g., carbon-based materials such as pharmaceuticals) due to low contrast with the epoxy, or even a possible chemical reaction between the sample and the epoxy. Size segregation, for example, by preferential settling of large particles during curing, is a concern, and it has been proposed that the epoxy plus powder mixture in the capillary should be agitated by shaking until the epoxy has set [29]. Furthermore it is not easy to discern if mixing with epoxy has actually separated all particles, or if small agglomerates have been adhered together. Because of this particle agglomeration, there might still be a need to subsequently separate particles digitally in the dataset. Alternatively, even though the agglomerates might be of interest in the characterisation, they can be excluded from quantification out of caution [40].

A different way of dispersing the particles is to spray them as a thin layer on a flat adhesive material, such as adhesive tape [40] or wax [37], which can then be rolled into a cylindrical shape for scanning (Figure 5c). The downsides of this method are like those due to mixing with epoxy, in that contrast might be low and an even distribution of particles on the material cannot easily be ascertained.

For samples in which particle arrangement needs to be preserved, a way of preparing samples is to impregnate a larger core or sample with resin, and then use a small core drill to extract samples with a diameter suitable for scanning [18]. Such an approach has the advantage of leaving the grains in their natural position, so that features such as grading of sand grains can be studied (Figure 5d).

A special case involves studies that place high priority on accurate shape description, such as those working on particle modelling. In such cases, individual particles are typically spaced manually inside a larger container, and supported in a high-viscosity matrix, for example, silicon oil [26,42,54].

### 2.2. Step 2: Data Acquisition and Reconstruction

X-ray CT data acquisition parameters, such as source energy and image exposure times, depend on the specific instrument, and a comparison is therefore not very informative. Of the 62 studies surveyed in this review, 29 reported laboratory X-ray source energy parameters. There was a wide range of source accelerating voltages, for example, quartz sand- and glass-containing samples were scanned at accelerating voltages over a large range: 25 kV to 150 kV. Exposure times for the projection images, which are dependent on the detector sensitivity, source parameters, sample attenuation, and the source-detector distance, are typically not explained in the method descriptions. In general, it must be assumed that scan data acquisition and reconstruction were carried out with settings that ensure good quality data [68]. Apart from the capabilities of the instrument, consideration must be given to the sample material composition and attenuation, the sample size, and the resolution required to image the particles. These parameters are discussed in more detail in Section 3.1 and Section 3.2 below.

### 2.3. Step 3: Image Pre-Processing

As image noise is an inherent feature of X-ray CT images [76], a noise-smoothing filter is typically applied as a first step. Smoothing of images not only suppresses localised deviations in brightness, which could be noise, but also sharpens peaks in the histogram, which helps with image segmentation [77]. Due to low computational costs, and wide implementation in image processing software, many authors apply simple median or Gaussian filters [23,24,32]. However, as those filters tend to blur edges, other researchers have employed bilateral [46] or non-local means filters [51,55], which have become more usable for large datasets with increasing computer performance. Instead of using an image-smoothing filter, some authors prefer to set a minimum voxel or volume cut-off limit to remove the smallest artefacts before quantification [52,58].

### 2.4. Step 4: Image Binarisation and Segmentation

Before quantification of particle size and shape is possible, the borders of each particle in the dataset must be identified and the particle given an individual number in a process called segmentation or labelling. The process usually begins with binarising the image into particles and background (which might be air or a surrounding medium such as epoxy), followed by separation and labelling of individual particles. For binarisation, typically a simple greyscale threshold is set or found with an algorithm such as Otsu’s method [78]. Thresholding can be difficult if there is a strong variation in brightness across the image or between particles, in which case a machine learning tool such as the trainable WEKA segmentation tool implemented in ImageJ [79] might provide better results [22]. If the particles are physically separated, such as when intentionally spaced apart, or separated by another medium such as epoxy, they can be labelled directly from the binary image by cluster detection [29]. However, in cases where particles are touching, such as when loose particles have been scanned in a capillary, particle boundaries fall below the resolution limit, and the particles may appear to be merged. An underlying assumption must be made that the particles are indeed separate and are not fused or cemented together. In these cases, additional image processing methods need to be employed to separate them into individual particles. The challenge is to find particle boundaries that preserve the actual shape of the particles, without breaking single particles into multiple ones, or merging multiple particles together.

**Figure 6:**
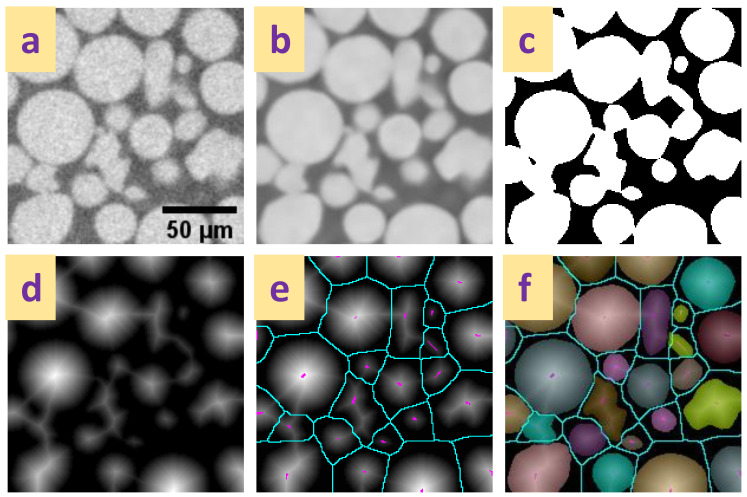
Particle separation process with a distance-transform watershed method. (**a**) Unprocessed image, (**b**) smoothed image (non-local means filter), and (**c**) binarised image, (**d**) distance-transform of the binary image shown in Figure 6c, (**e**) distance transform with the extended maxima markers (purple) and watershed lines (blue) shown, (**f**) segmented particles resulting from re-flooding the image from the markers to the boundaries of the binary mask image and the watershed lines.

**Figure 7:**
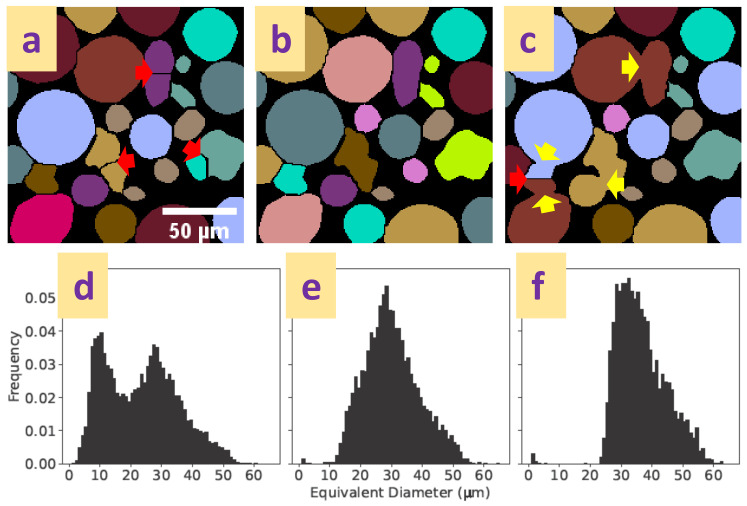
Segmentation errors and their effect on the particle size distribution of a copper powder with a manufacturer’s size range of 15–45 μm. Top row: (**a**) over-segmentation with increased splitting of particles (red arrows), (**b**) visually correct segmentation, (**c**) under-segmentation with increased merging of particles (yellow arrows). Segmentation differences are a result of varying the extrema marker extend (Figure 6e) during the watershed process. Segmentation with the open-source ImageJ plugin MorphoLibJ [82]. Bottom row: Particle size distributions for the full datasets: (**d**) 8800 particles (**e**) 5700 particles, (**f**) 3300 particles resulting from the segmentations shown in top row (**a**–**c**).

A common approach is to use a distance transform-based watershed to separate particles, illustrated in Figure 6. A distance map of the particle phase is calculated by successive erosion of the border of particles, the centres of the particles identified and labelled as markers, and the final label image created by re-flooding the binary image by a watershed process [77,80]. While this approach works very well for spherical, equant particles, complex particle shapes present additional challenges. For example, the erosion process of complex particle shapes often leads to multiple central spots, which, if uncorrected, result in over-segmentation by splitting whole irregular particles into multiple parts. A range of approaches exist to correct the marker image, for example, by eliminating weak markers with an h-extrema filter [81] or by stopping the erosion early [60]. Removing too many markers results in under-segmentation and the artificial merging of separate particles. Figure 7 illustrates over- and under-segmentation, and the resulting particle size distributions. Care evidently must be taken to choose appropriate watershed parameters, and human supervision of the process is recommended. Unsupervised algorithms can be used to evaluate the segmentation if a particular particle shape is expected and can be used as a quality marker [39].

### 2.5. Step 5: Measurements and Quantification

Once particles have been separated in a satisfactory way, each particle can be measured. Table 1 presents an overview of the most commonly employed size and shape parameters to describe particles.

While each particle is originally represented by a cluster of voxels in the segmented 3D dataset, it can be advantageous for data storage demands and processing speeds to approximate the particle surface with either a triangular surface mesh [83] or with a series of spherical harmonics (SH) functions [2,27]. Approximating the particle surface in either of those ways also introduces a degree of surface smoothing, which can help deal with unrealistic effects of surface voxelisation. However, it is difficult to use SH functions to describe particles with more complex shapes, for example, when the centre of gravity lies outside of the particle [52]. Most morphological parameters can be calculated directly from the voxelised representation, although it can be faster to calculate them from the mesh or SH approximation of the particle. Table 1 lists the alternative approaches to calculate each measure. Currently, no agreement over the best approach exists, and various software solutions implement one or more of the methods. While, in general, results from different approaches should be similar, small deviations of results exist due to the different approximations employed, which might limit the usefulness of specific approaches [46]. This especially affects surface area measurements. Simply counting the faces of the surface voxels usually leads to overestimation of the surface area, and approximation approaches with surface meshes, SH functions, or algebraic estimation [84,85] give more realistic results [86].

The 3D data allow the measurement of the length (L), width (W), and breadth (B), also called depth or thickness, of each particle, which again can be found in multiple ways, but most commonly by determining the principal axes of the inertia tensor and computing the moments of inertia (with mass represented by voxel intensity) [87]. Knowing the three dimensions L, W, and B, can improve the comparison with other particle measurement methods; for example, one study found that the width of a particle correlated well with sieve analysis data, while the length correlated well with laser diffraction data [7]. The dimensions also allow calculation of the aspect ratios, which can be used to classify the particles, for example, by the four Zingg classes [66] of discs, spheres, blades, and rods (Figure 2b).

Apart from the morphological parameters mentioned in Table 1, many other parameters can be calculated; for example, particle projections at different angles [52] can be directly compared with 2D measurements, e.g., from microscopy images. Careful analysis of the surface curvature allows for determination of particle roundness in 3D [26,42,75], which is more difficult than in 2D [88], and thus not commonly implemented in analysis software.

In addition to measuring each particle, properties of the granular assembly, such as the packing density or bulk porosity can also be quantified, if the sample was prepared in a bulk state (and not diluted by, for example, epoxy).

Resulting measurement data are usually presented in tabular form or summarised statistically. However, it is often also possible to visualise results by combining them graphically with image data, for example, as colour coding of the original 3D data (Figure 2).

**Table 1:** Overview of common particle size measures, and their methods of calculation from the digital image data. The same measure can often be calculated in multiple ways, as listed in the right-hand column. SH refers to approximating the particle surface with spherical harmonics functions.

Sketch	Measure	Methods of Calculation
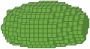	**Volume**	Counting of all voxels belonging to a particle [89], integral over SH functions [87], integral over the surface covered by a mesh [90].
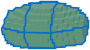	**Surface Area**	Counting all faces of surface voxels, estimation of the surface area [84,85], measuring a surface mesh (marching cubes [90]), or calculated from the SH functions [87].
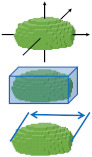	Three dimensions of the particle—**length (L), width (W), and breadth (B)** (also called depth or thickness). These are mutually orthogonal and L ≥ W ≥ B	Derived from the moments of inertia (with mass represented by voxel intensity) [52,87], edge length of the smallest box that contains the particle [91,92], searching the SH parameters [52], or by calculating length as the maximum Feret [93] or caliper diameter, the maximum distance between two tangential planes of the particle surface and finding W and B orthogonally [94].
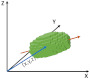	**Position** of the particle within the dataset	Centroid (centre of mass) position [89], as the origin of a square box containing the particle, or as the first point of the particle encountered in the searching direction.
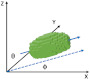	**Orientation** of principal axes, ϕ, θ	Principal axis orientation derived from moments of inertia (or volume) tensor [87].
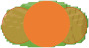	**Local Thickness**	The diameter of the largest sphere that fits inside the particle at a local point. [95]. The local thickness differs from the total thickness especially in cases of porous or cup-shaped particles.
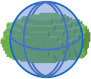	**Equivalent Diameter** of a sphere of the same volume as the particle	Derived measure from volume (*V*): Equivalent diameter $=\sqrt[{3}]{6V/\pi }$
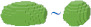	**Sphericity** measures between 0 and 1, and shows how closely the shape matches a perfect sphere	Derived measure from volume (*V*) and surface area (*A*): Sphericity $=\sqrt[{3}]{(36\pi V^{2})/A}$

## 3. Outlook and Limits of the Method

After here summarising the range of work already undertaken, two research questions with regards to the limits of particle characterisation by X-ray CT will be subsequently discussed. Following the development of sub-micrometre commercial X-ray CT systems, the first question concerns the smallest particle size that can be successfully characterised using laboratory equipment. The second question concerns the types of materials that can be used for powder analysis using X-ray CT, especially regarding highly X-ray attenuating materials.

### 3.1. Limits of Particle Size and Resolution

Currently, the highest resolving commercial X-ray CT systems advertise a sub-micrometre resolution, as small as 0.5 micrometres. This limit results from hardware parameters, such as the source spot size and the physical detector resolution [68]. The definition of 3D spatial resolution in X-ray CT is complicated, and a topic of current debate [96]. The 2D resolution of each projection can be measured with resolution targets such as the JIMA (Japan Inspection Instruments Manufacturers’ Association) chart [97] or by evaluation of the modulation transfer function [98]. However, the true 3D resolution can vary from scan to scan due to additional factors such as the number of projections, the reconstruction algorithm, the complex energy spectrum, and the sample shape relationship. Standardised measurement test samples (phantoms) and defining standards for 3D resolution are under active development [96]. In the following section, it is assumed that the approximate highest resolution is 0.5 μm. It should be noted that, in most X-ray CT systems, the pixel size (and resulting voxel size) does not equal the spatial resolution of the system at the current conditions. Firstly, it is typically possible to decrease the pixel size significantly to below the current spatial resolution by increasing the magnification (oversampling). Secondly, according to the Nyquist–Shannon sampling theorem, the sampling rate must be at least two times greater than the signal, which means at least two pixels are needed to detect a feature. For example, to achieve a spatial resolution of 0.5 μm, a pixel size of at least 0.25 μm should be used.

To characterise loose particles, three conditions must be met: the particle must be observable over the resolution limit, it must be separate from neighbouring particles, and it must be made up of enough voxels to describe its size and shape.

Fewer voxels are needed if the absorption contrast to the surrounding medium is high and only the general position needs to be determined. In the case of spaced-apart particles, two voxels are enough to identify a particle. With more densely packed particles, this identification is more difficult, because the size of gaps between the particles typically becomes the limiting factor. This is especially true in case of spherical particles, where the convex shape reduces the gap between particles to less than the resolution limit. In such cases, enough of the particle must be without surface contact to enable separation from its neighbours by, e.g., a watershed process (Section 2.4). Broad particle size distributions (e.g., in poorly sorted samples), where smaller particles fill the gap between larger ones, make this separation even more challenging.

It is straightforward to appreciate that more voxels per particle result in a more accurate shape description, but the question is how few voxels could be considered to be enough? In practice, often a voxel, or volume, cut-off limit is defined, under which particles are not further evaluated, even though this can affect the resulting particle size distribution. Commonly, 512 voxels are used for particles separated in epoxy [52], which is an 8 × 8 × 8 cube, or a sphere with a diameter of 10 voxels. Other studies have used a much lower limit, such as 8 or 10 voxels [37,45]. Assuming a sub-micrometre resolution X-ray CT system, an approximate smallest voxel size at the highest resolution is approximately 0.2 to 0.3 μm. Using the 512 voxel cut-off limit, this means the smallest characterisable particle size is approximately 2 to 3 μm. However, calculating the average amount of voxels across the smallest particles, in our survey of published research [1,2,3,4,5,6,7,8,9,10,11,12,13,14,15,16,17,18,19,20,21,22,23,24,25,26,27,28,29,30,31,32,33,34,35,36,37,38,39,40,41,42,43,44,45,46,47,48,49,50,51,52,53,54,55,56,57,58,59,60,61], has revealed that, in most cases, approximately 25 voxels are used, which equates to the smallest characterisable particle size being 5 μm. A small voxel size necessitates a similarly small field of view (of, e.g., 500 × 500 μm for a 0.25 μm voxel size with a 2000 × 2000 pixel detector), and very high-resolution scans might not be feasible if the sample size cannot be sufficiently reduced, or if insufficient numbers of larger particles can fit into the field of view. Many commercial X-ray CT systems also cannot achieve their highest resolution at high power [68], mainly due to source point spread with increasing power [99] and increased detector blurring [100], which limits the materials that can be analysed at high resolution.

### 3.2. Limits of Material Suitability

**Figure 8:**
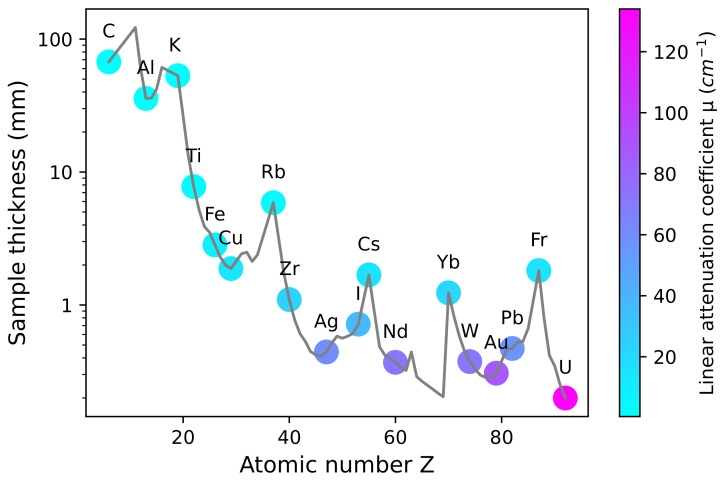
Estimation of sample thickness for a loose particle sample in a capillary for a material with atomic number Z. Calculations assume a packing density of 60%, an X-ray transmission of 20% and an effective energy of 60 keV [101], for which mass attenuation coefficients μ/ρ have been taken from in the National Institute of Standards and Technology’s (NIST, USA) X-ray Mass Attenuation Coefficients database [102]. The grey line shows estimated sample thickness for all elements (Z = 8 to Z = 92, excluding gases), while the coloured dots highlight selected elements. The colour represents the linear attenuation coefficient, μ, calculated from the mass attenuation coefficient μ/ρ and the density ρ.

While X-ray CT analysis has been applied successfully to characterise particles made from a wide range of materials, a limiting factor is the requirement for X-ray transmission through the sample, given that X-ray transmission reduces with increasing atomic number. The higher the atomic number, the thinner a sample has to be for sufficient X-ray transmission, assuming constant energy of the beam (Figure 8). In the following section, we will establish the approximate thickness of single-element samples that is possible to scan with a typical laboratory X-ray CT instruments. In principle, the thickness *t* for a given transmission $I/I_{0}$ can be calculated by re-arranging the Lambert–Beer equation:

(1) $$I=I_{0}e^{-\frac{\mu }{\rho }\cdot \rho t}\Leftrightarrow t=1/\mu \cdot ln\left(I_{0}/I\right)$$

with *I* intensity, *I*${}_{0}$ initial intensity, ρ density, and μ/ρ mass attenuation coefficient. The Lambert–Beer equation assumes a monochromatic, parallel beam and single-material sample of constant thickness. Those assumptions are not met in the reality of laboratory X-ray sources with polychromatic and divergant beams, detectors with unknown energy response functions, and multi-material, irregularly shaped samples. However, an approximation of the sample thickness can still be made to understand the limits of the current methodology.

Most modern laboratory X-ray CT systems operate with a polychromatic source, often with a tuneable accelerating voltage up to 160 kV or 225 kV. However, because of the nature of the bremsstrahlung spectrum, the proportion of high-energy photons within the spectrum is very small. As a first step in simplifying the problem, the polychromatic spectrum can be approximated by a single effective energy [101]. This energy is typically considerably lower than the maximum possible energy for the system; for example, the effective energy of a 225 kV tungsten target source has been calculated to be between 40 kV and 80 kV, depending on accelerating voltage [101]. Once a suitable energy has been estimated, the mass attenuation coefficient and the density for a given material for that energy can be found from tabulated values [102,103]. Further assuming a collimated X-ray beam and a flat sample (or the centre of a cylinder), the thickness *t* for a given transmission can be calculated with the Lambert–Beer equation above (Equation (1)). To derive Figure 8, an effective energy of 60 keV has been assumed, along with a transmission of 20%. Additionally, it has been assumed that the sample is a granular material with a packing density of 60%. Samples made of materials with atomic numbers higher than 40 (zirconium) would have to be thinner than <1 mm to achieve sufficient transmission. As samples under 1 mm become difficult to handle in practice, an alternative way of dealing with particles of high-Z materials is to disperse them in epoxy resin to achieve a reduced packing density. This, in turn, allows for thicker samples of an equivalent X-ray transmission.

### 3.3. Outlook and Future Developments

As this review has shown, particle analysis with X-ray CT has become an often-used methodology. However, there is scope for the method to become more widely used, as, thus far, the majority of publications citing the use of this technique stem from additive manufacturing, with only a few examples from other disciplines, such as geological studies or pharmaceutical development. Particle size, if over a few micrometres, and material composition are not necessarily limiting factors, as long as the samples are adequately prepared. The non-destructive nature of X-ray imaging, along with the potential to prepare samples without the need to embed them in resin, makes the method especially suitable for substances that might react with epoxy, or have a similar composition to epoxy, which would decrease the contrast.

Although considerable increases in spatial resolution of laboratory X-ray CT instruments seems to be unlikely in the near future, improvements might be possible with regards to the field of view. The development of larger detectors, or of projection image stitching methods, would allow for a larger FoV with a similar voxel size. Especially for spaced-apart particles, a larger FoV would enable scans to simultaneously cover a larger quantity of particles. Currently, a limiting factor with regards to image size, is the magnitude of the resulting data files. However, with increasing computer memory capacity, this limitation is likely to be overcome. Further improvements are also likely with regards to noise and other image artifact reduction, through improved image filters and machine learning routines. This would enable clearer segmentation, especially of closely packed particles, which is currently a limiting factor for loose particle samples.

## 4. Conclusions

1 Particle characterisation with X-ray CT has become a widely used method over the last 20 years.
2 The advantages of X-ray CT are the ease of sample preparation, and the available measures of the 3D size and morphology of the particles, as well as internal features such as intra-particle porosity and sample heterogeneity.
3 Since each X-ray CT scan typically encompasses tens of thousands of particles, it is easy to achieve statistically significant results.
4 Modern sub-micrometre X-ray CT systems are able to scan particles as small as 5 µm, or potentially as small as 2 to 3 µm, if the particles are spaced apart.
5 Using theoretical approximations, we have shown that X-ray CT is suitable for characterising materials with atomic numbers up to Z = 40 when the sample is prepared in form of loose particles in a capillary.
6 Materials with an atomic number greater than 40 need special sample preparation methods such as diluting in epoxy in order to achieve enough X-ray transmission from a typical laboratory source.

## Data Availability

Not applicable.
